# Curcumin Supplementation Ameliorates Bile Cholesterol Supersaturation in Hamsters by Modulating Gut Microbiota and Cholesterol Absorption

**DOI:** 10.3390/nu14091828

**Published:** 2022-04-27

**Authors:** Ting Hong, Jun Zou, Xin Jiang, Jie Yang, Zhuo Cao, Youming He, Dan Feng

**Affiliations:** 1 Guangdong Provincial Key Laboratory of Food, Nutrition and Health, Department of Nutrition, School of Public Health, Sun Yat-sen University, Guangzhou 510080, China; hongt3@mail2.sysu.edu.cn (T.H.); jiangx67@mail2.sysu.edu.cn (X.J.); yangj575@mail2.sysu.edu.cn (J.Y.); caozh27@mail2.sysu.edu.cn (Z.C.); heym36@mail2.sysu.edu.cn (Y.H.); 2 Guangzhou Key Laboratory of Environmental Pollution and Health Risk Assessment, Department of Occupational and Environmental Health, School of Public Health, Sun Yat-sen University, Guangzhou 510080, China; 3 Department of Cardiology, The Sixth Affiliated Hospital, School of Medicine, South China University of Technology, Foshan 528200, China; zoujun961@163.com

**Keywords:** curcumin, gut microbiota, cholesterol absorption, cholesterol supersaturation, gallstones

## Abstract

Curcumin is a polyphenol that has been shown to have prebiotic and cholesterol-lowering properties. This study aimed to investigate the impact of curcumin on bile cholesterol supersaturation and the potential mechanistic role of intestinal microbiota and cholesterol absorption. Male hamsters (*n* = 8) were fed a high-fat diet (HFD) supplemented with or without curcumin for 12 weeks. Results showed that curcumin significantly decreased cholesterol levels in the serum (from 5.10 to 4.10 mmol/L) and liver (from 64.60 to 47.72 nmol/mg protein) in HFD-fed hamsters and reduced the bile cholesterol saturation index (CSI) from 1.64 to 1.08 due to the beneficial modifications in the concentration of total bile acids (BAs), phospholipids and cholesterol (*p* < 0.05). Gut microbiota analysis via 16S rRNA sequencing revealed that curcumin modulated gut microbiota, predominantly increasing microbiota associated with BA metabolism and short-chain fatty acid production, which subsequently up-regulated the expression of hepatic cholesterol 7-alpha hydroxylase and increased the synthesis of bile acids (*p* < 0.05). Furthermore, curcumin significantly down-regulated the expression of intestinal Niemann–Pick C1-like protein 1(NPC1L1) in hamsters and reduced cholesterol absorption in Caco-2 cells (*p* < 0.05). Our results demonstrate that dietary curcumin has the potential to prevent bile cholesterol supersaturation through modulating the gut microbiota and inhibiting intestinal cholesterol absorption.

## 1. Introduction

Gallstone disease (GD) is a common and frequent digestive disease caused by an imbalance of bile components, and most gallstones are gallbladder cholesterol stones [1]. The formation of gallstones is closely related to abnormal cholesterol metabolism, and the supersaturation of biliary cholesterol has been reported to be a prerequisite of cholelithiasis [2]. Cholesterol saturation index (CSI) is known as the most reliable indicator of the cholesterol supersaturation and crystals in gallbladder bile [3].

The gut microbiota is considered as a metabolic organ for its important role in the host health [4]. Available evidence suggests that the intestinal flora is associated with the formation of gallstones [5]. It has been reported that compared with heathy people, the diversity and abundance of intestinal bacteria were altered in the patients with gallstones [6]. Wang et al. found that the lithogeny diet (LD) reshaped the gut flora composition and resulted in cholesterol gallstones in C57BL/6J mice [7]. Fremont-Rahl et al. demonstrated that alterations in the gut flora can affect the pathogenesis and the susceptibility of cholesterol gallstones [8]. Altered bile acid (BA) metabolism or elevated cholesterol levels in the bile are known as direct causes of cholesterol stones. The intestinal flora plays an indispensable role in the metabolism of BAs [9], and some gut flora BAs metabolites have been shown to regulate cholesterol 7-alpha hydroxylase (CYP7A1) and affect bile cholesterol conversion [10]. In addition, short-chain fatty acids (SCFAs), products of bacterial fermentation of indigestible carbohydrates, have been reported to possess cholesterol-lowering potential via inhibiting hepatic cholesterol synthesis and promoting the excretion of BAs [11,12,13].

Polyphenols extracted from natural plants have received widespread attention for their health-promoting benefits in recent years [14]. Curcumin is a polyphenol extracted from turmeric that exhibits anti-inflammatory, anti-oxidative and cholesterol-lowering effects [15,16]. Researchers found that curcumin treatment reduced the formation of gallstones in mice, an effect attributed to curcumin’s cholesterol-lowering and promoting gallbladder emptying properties [17,18,19]. Our previous study found that curcumin can reduce intestinal cholesterol absorption by down-regulating the expression of Niemann–Pick C1-like protein 1 (NPC1L1) [16,20], which is a transmembrane protein responsible for promoting cholesterol absorption in the intestine. Recently, curcumin has been considered as a potential prebiotic that promotes the growth of probiotic bacteria such as *Lactobacillus* and *Bifidobacterium* [21]. However, as a potential prebiotic and cholesterol absorption inhibitor, the relationship between curcumin’s anti-lithogenic activity and gut microbiota and intestinal cholesterol absorption has not been elucidated.

Based on the above information, we speculated that curcumin treatment could modulate gut microbiota composition and cholesterol absorption, improve cholesterol and BA metabolism, ameliorate bile cholesterol supersaturation and finally reduce the formation of gallstones. In this study, male Syrian golden hamsters were fed a high-fat diet (HFD) supplemented with or without curcumin for 12 weeks to test our hypothesis.

## 2. Materials and Methods

### 2.1. Animals and Treatments

The 5-week-old male Syrian golden hamsters were purchased from Baineng Biotechnology Co., Ltd. (Guangzhou, China). All hamsters were specific-pathogen-free (SPF) and were fostered in a barrier environment (12 h light/dark cycle; temperature at 22 °C). One week after acclimation to the environment, the sample function of R software (V2.15.3, R Core Team, Vienna, Austria) was used to generate random numbers and all hamsters were randomly divided into two groups (8 hamsters/group) fed with HFD (containing 0.15% cholesterol; 41% of energy from fat) or HFD added with curcumin (0.1% w/w) (Sigma-Aldrich, St. Louis, MO, USA). During the experiment, hamsters were weighed weekly. At the end of the 12-week experiment, the feces of the hamsters were collected for three consecutive days. All hamsters were executed under anesthesia after overnight fasting. Orbital blood was centrifuged to collect serum. The liver and gallbladder were photographed. The entire gallbladder wall and bile were collected separately. The liver and intestine were excised for histological and biochemical analysis. The remaining tissues were frozen at −80 °C.

**Figure 1:**
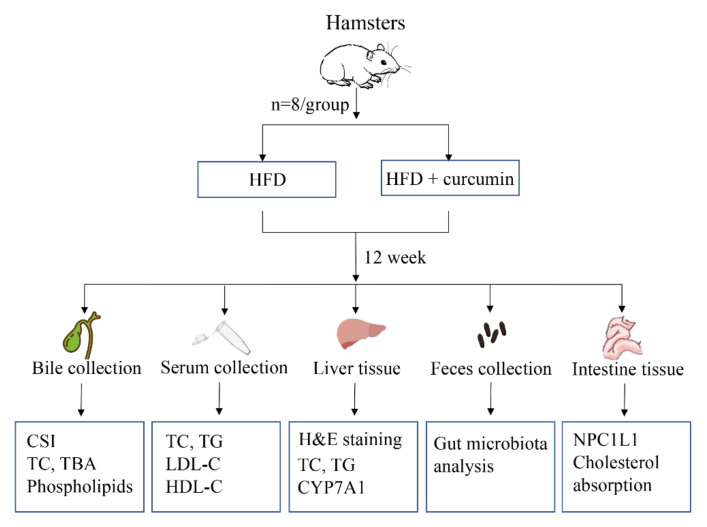
Experimental procedure. HFD, High-fat diet; CSI, Cholesterol saturation index; TC, Total cholesterol; TBA, Total bile acids; TG, Total triglycerides; LDL-C, Low-density lipoprotein cholesterol; HDL-C, High-density lipo-protein cholesterol; CYP7A1, Cholesterol 7-alpha hydroxylase; NPC1L1, Niemann–Pick C1-like protein 1.

The present study primarily investigated whether curcumin supplementation reduced bile cholesterol saturation index (CSI) in hamsters fed an HFD. Since bile cholesterol saturation is closely related to cholesterol metabolism, serum and liver lipid levels were also investigated. The underlying mechanism was further investigated. Gut microbiota analysis was conducted to explore the role of gut microbiota in the effect of curcumin on cholesterol saturation. We further measured the expression of hepatic CYP7A1 as gut flora metabolites can regulate CYP7A1 through signaling pathways, thus affecting bile cholesterol conversion. In addition, we examined the effect of curcumin on intestinal cholesterol absorption by measuring the expression of intestinal NPC1L1 protein in hamsters and cholesterol absorption in Caco-2 cells. The experimental procedure is shown in Figure 1.

### 2.2. Histological Analysis

The parts of the liver tissue containing the portal veins were fixed in 4% paraformaldehyde solution for 24 h, embedded in paraffin and sectioned (5 μm) for hematoxylin-eosin (H&E) staining. Sections were observed under a microscope, and the clear and typical field of view was captured.

### 2.3. Biochemical Analysis

Serum total cholesterol (TC), total triglycerides (TG), low-density lipoprotein cholesterol (LDL-C) and high-density lipoprotein cholesterol (HDL-C) concentrations as well as liver TC and TG contents were measured using commercial assay kits (Jiancheng, Nanjing, China) according to the instructions of assay kits. Bile total bile acids (TBA), TC levels were determined by assay kits (Jiancheng, Nanjing, China) and bile phospholipids was detected by assay kits from Hengyuan Biotech Co., Ltd. (Shanghai, China). The total lipid concentration and phospholipid/ (phospholipids plus TBA) ratio were used to calculate CSI according to Carey’s critical tables.

### 2.4. Real-Time Polymerase Chain Reaction (RT-qPCR)

A total of 20 mg of liver tissue was taken to extract RNA using the TRIzol reagent (Thermo Fisher, Waltham, MA, USA). The dried RNA precipitate was dissolved in DEPC water and then tested for RNA concentration and purity using NanoDrop^®^ (NanoDrop Technologies Inc., Wilmington, DE, USA). ReverTra Ace^®^ RT-qPCR Kit (Toyobo, Osaka, Japan) was purchased and the RNA samples were reverse transcribed to cDNA according to the instruction and stored at −20 °C. Primers were obtained from Sangon Co. Ltd. (Shanghai, China). The primer sequences of CYP7A1 are as follows: Forward sequence: AAGACGACATCATCGCTCTTTA; reverse sequence: ACAATCCCTATGGCTTCACTTT. Realtime PCR Master Mix (Toyobo, Osaka, Japan) was performed using the Applied Biosystems Real-Time 7500 System (Thermo Fisher Scientific Inc., Waltham, MA, USA), and the results were calculated using the 2^−ΔΔCT^ method. β-actin served as a reference gene for the relative expression level of CYP7A1.

### 2.5. Western Blot

A total of 20 mg of liver tissue or 1 cm intestine was homogenized in 400 μL of lysate, and then the protein concentration was determined by the BCA Protein Assay Kit (Beyotime, Shanghai, China). After adding 5× loading buffer to the protein samples at a ratio of 4:1, the protein lysates were denatured at 100 °C for 5 min. The polyacrylamide gel was configured and then subjected to the steps of electrophoresis, membrane transfer, blocking, incubation with primary antibodies (anti-NPC1L1, anti-CYP7A1 and anti-β-actin, Abcam, Cambridge, MA, USA) and secondary antibody (Goat Anti-Rabbit IgG-HRP, Abcam, Cambridge, MA, USA). Finally, the strips were dripped with ECL luminescent solution (Thermo Fisher, Waltham, MA, USA), exposed under an automated chemiluminescent imaging analysis system, and quantified using Image J software 1.8.0 (NIH, Bethesda, MD, USA).

### 2.6. Gut Microbiota Analysis

Feces were selected to extract the bacterial DNA according to the instructions of TIANamp Stool DNA Kit (Tiangen Biotech, Beijing, China). Primers (Invitrogen, Carlsbad, CA, USA) containing 515F and 806R (515F: 5′-GTGCCAGCMGCCGCGGTAA-3′; 806R: 5′-GGACTACHVGGGTWTCTAAT-3′) were applied for PCR amplification of the 16S rDNA V4 region. An EZNA Gel Extraction Kit (Omega, La Chaux-de-Fonds, Switzerland) was used to get a mixture of purified PCR products. A NEBNext^®^ Ultra™ DNA Library Prep Kit for Illumina^®^ (New England Biolabs, Beverly, MA, USA) was adopted to build Sequencing libraries, and then the data were sequenced using the Illumina Hiseq2500 platform. The operational taxonomic unit (OUT) was obtained by sequence clustering analysis (sequences with similarity ≥ 97%). α-diversity indexes including richness, Shannon index and Simpson index were analyzed by QIIME alpha_diversity.py (V1.9.1, http://qiime.org/scripts/alpha_diversity.html, accessed on 26 March 2022) and R software (V2.15.3, R Core Team, Vienna, Austria). Larger Shannon index values and smaller Simpson index values implied higher community diversity. β-diversity analysis indicates the difference of species complexity between different samples. Bray–Curtis and unweighted unifrac distance of β-diversity index were obtained with QIIME software. Based on the Bray–Curtis unifrac distance and unweighted unifrac distance, non-metric multidimensional scaling (NMDS) plot and the sample distance heatmap were drawn with R software (V2.15.3 R Core Team, Vienna, Austria).

### 2.7. Cholesterol Absorption Assay

The human colon cancer cell lines Caco-2 were obtained from Fuheng Biological Cell Bank (Shanghai, China) and maintained in high-glucose DMEM medium (Gibco, NY, USA). Cells at approximately 60% density were treated with or without 12 μmol/L curcumin for 24 h, and then incubated with 4 μmol/L 22-NBD-cholesterol for 4 h. The cells were observed by an inverted fluorescent microscope (Leica, Zeiss, Germany) and typical images were captured. To determine the fluorescence intensity of 22-NBD-cholesterol in cells, a fluorescence spectrophotometer (SpectraMax M5, Molecular Devices Corporation, San Jose, CA, USA) was adopted at 469 nm excitation and 537 nm emission wavelengths. The results of cholesterol absorption were expressed as fluorescence intensity/mg protein.

### 2.8. Statistics

To calculate the minimum size of the study group, we used the degree of freedom(E) of variance analysis. E is equal to the number of total experimental animals minus the number of groups and its value should be between 10 and 20. According to this formula and taking into account animal mortality, 8 hamsters were assigned to each group.

All results were expressed as mean ± standard error of mean (SEM). SPSS 25.0 (SPSS Inc., Chicago, IL, USA) and GraphPad Prim 8.0 software (GraphPad Software LLC., San Diego, CA, USA) were used for statistical analysis and drawing, respectively. The Shapiro–Wilk test was used for the normality test and the Levene test was adopted to analyze the variance homogeneity. The difference between the two groups was assessed by *t*-test or non-parametric test. Spearman correlation analysis was used to analyze the relationship between the relative abundance of gut microbiota and biochemical parameters. *p* < 0.05 was defined as a significant difference. All data analysis was performed by SPSS 25.0.

## 3. Results

### 3.1. Curcumin Improved Bile Cholesterol Supersaturation in HFD-Fed Hamsters

**Figure 2:**
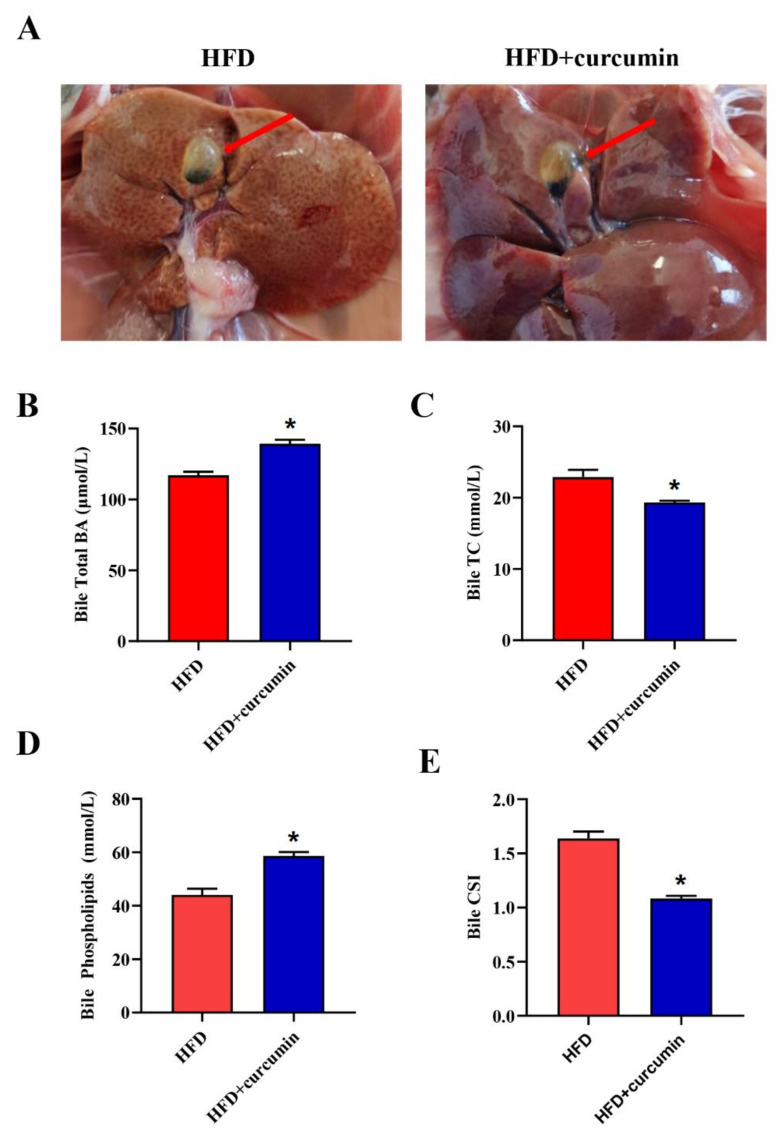
Effects of curcumin on bile compositions in gallbladder of HFD-fed hamsters. (**A**) Typical pictures of gallbladders; (**B**–**E**) The bile total BAs, TC, phospholipids levels and CSI in two groups. The red arrows refer to gallbladders. Data are expressed as mean ± SEM (*n* = 8), * *p* < 0.05 compared to HFD group. BAs, Bile acids; TC, Total cholesterol; CSI, Cholesterol saturation index.

No obvious cholesterol gallstones were found in either group of hamsters. However, unlike the curcumin-treated group whose gallbladders appeared clear and transparent, the HFD-fed group’s gallbladders appeared more turbid (Figure 2A). Furthermore, curcumin supplementation markedly increased bile TBA and phospholipid levels and decreased bile TC concentration, consequently decreasing the bile CSI from 1.64 to 1.08 in HFD-fed hamsters (*p* < 0.05) (Figure 2B–E).

### 3.2. Curcumin Ameliorated Serum and Liver Lipid Levels in HFD-Fed Hamsters

**Figure 3:**
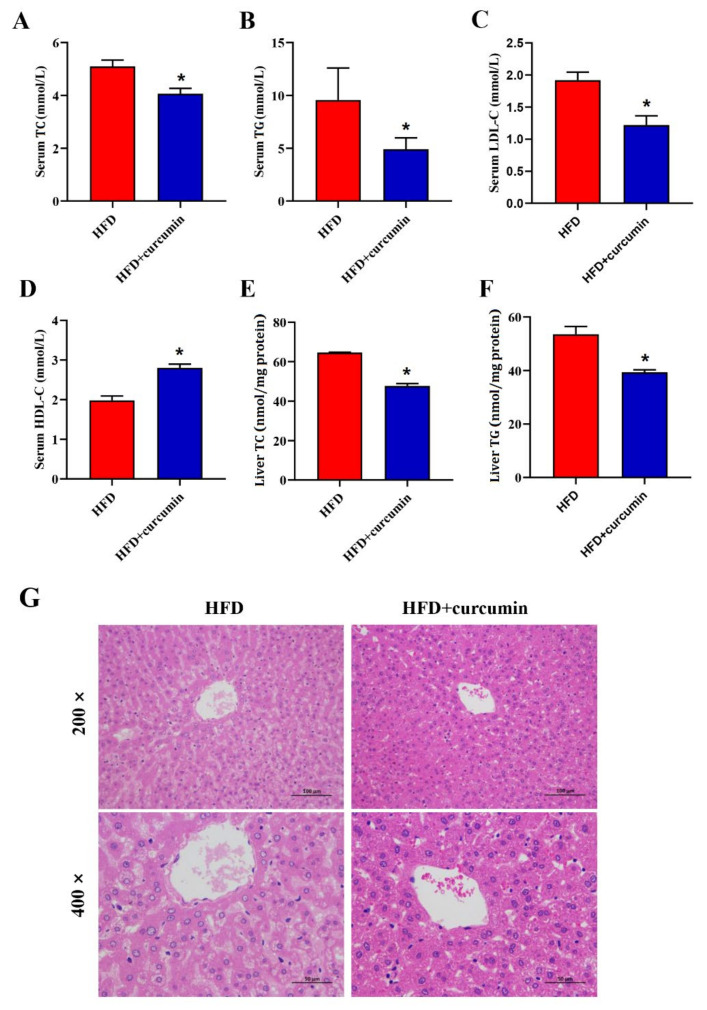
Effects of curcumin on serum and liver lipid metabolism in HFD-fed hamsters. (**A**–**D**) The serum TC, TG, LDL-C, HDL-C levels; (**E**,**F**) The liver TC, TG levels; (**G**) Representative H&E staining graphs of liver (200× magnifications, 400× magnifications). Data are expressed as mean ± SEM (*n* = 8), * *p* < 0.05 compared to HFD group. TC, Total cholesterol; TG, Total triglycerides; LDL-C, Low-density lipoprotein cholesterol; HDL-C, High-density lipoprotein cholesterol.

Administration of curcumin significantly reduced the levels of serum TC, TG, LDL-C and increased the level of serum HDL-C in hamsters fed HFD diet (Figure 3A–D). Simultaneously, dietary curcumin addition apparently lowered liver TG and TC contents (Figure 3E,F). Furthermore, the H&E staining pictures of liver demonstrated that curcumin noticeably alleviated hepatic fat accumulation induced by HFD (Figure 3G).

### 3.3. Curcumin Modulated Gut Microbiota Composition in HFD-Fed Hamsters

**Figure 4:**
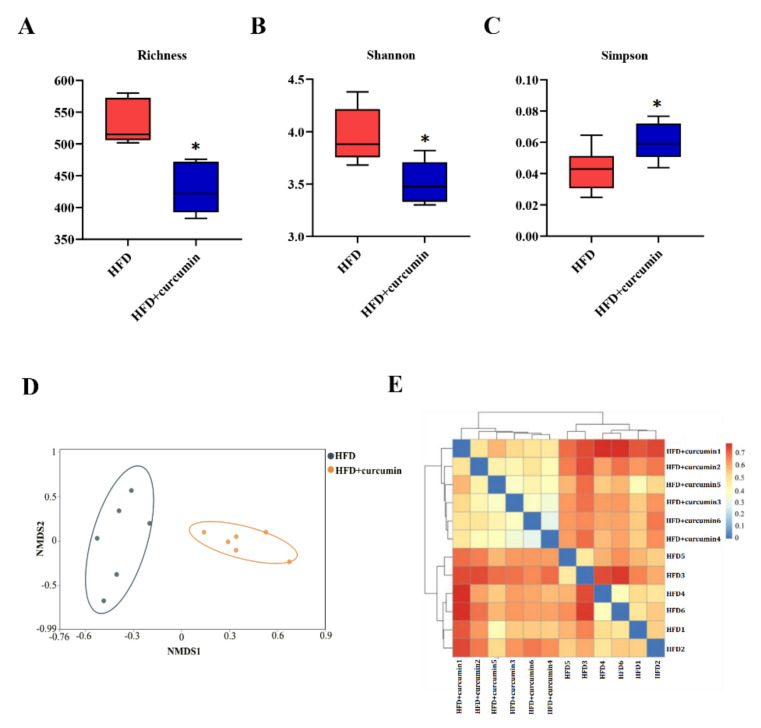
Effects of curcumin on gut microbiota composition in HFD-fed hamsters. (**A**–**C**) α-diversity analysis including richness, Shannon index and Simpson index of gut microbiota. (**D**,**E**) β-diversity analysis of gut microbiota including NMDS plot and sample distance heat map. Data are expressed as mean ± SEM (*n* = 6), * *p* < 0.05 compared to HFD group.

In the curcumin-treated group, the richness of gut microbiota was significantly lower than that in HFD group (*p* < 0.05) (Figure 4A). Meanwhile, the diversity of gut flora was decreased by curcumin addition, including the decreased Shannon index and the increased Simpson index (*p* < 0.05) (Figure 4B,C). Moreover, the NMDS plot and sample distance heatmap revealed a distinct divergence in intestinal flora composition between the two groups (Figure 4D,E).

### 3.4. Curcumin Modulated Gut Microbiota Abundance in HFD-Fed Hamsters

**Figure 5:**
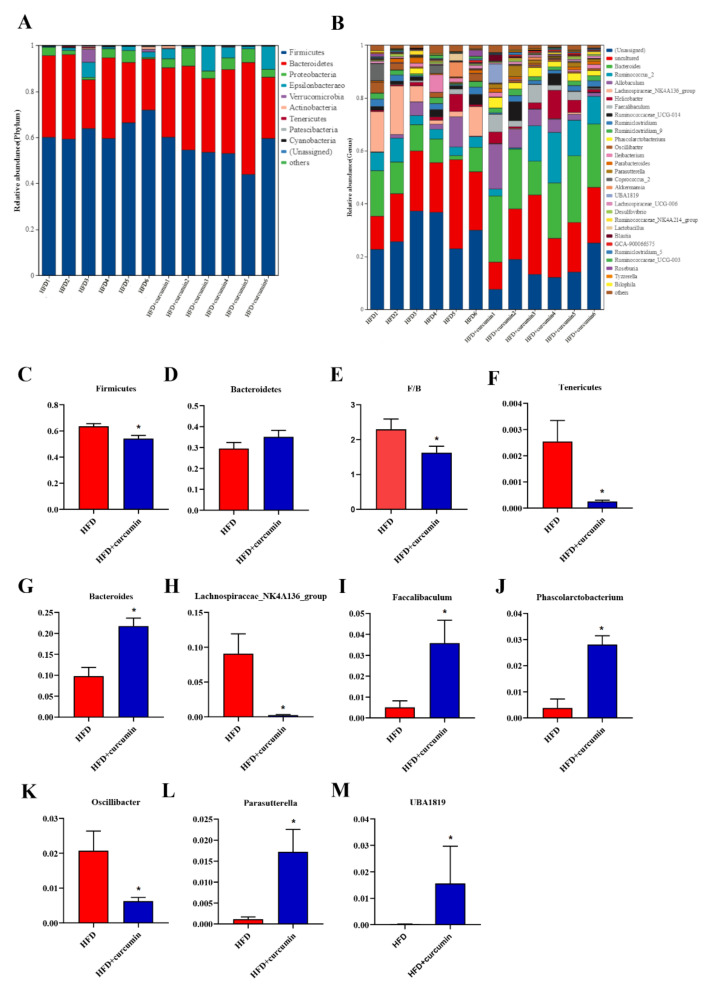
Effects of curcumin on gut microbiota abundance in HFD-fed hamsters. (**A**) Composition of gut microbiota at phylum level; (**B**) Composition of gut microbiota at genus level; (**C**,**D**) The relative abundance of *Firmicutes*, *Bacteroidetes*; (**E**) The ratio of *firmicutes* to *Bacteroidetes*; (**F**) The relative abundance of *Tenericutes*; (**G**–**M**) The relative abundance of *Bacteroides*, *Lachnospiraceae_NK4A136_group*, *Faecalibaculum*, *Phascolarctobacterium*, *Oscillibacter*, *Parasutterella* and *UBA1819*. Data are expressed as mean ± SEM (*n* = 6), * *p* < 0.05 compared to HFD group.

To further analyze the alterations in gut microbiota composition, the relative abundance of the main intestinal flora was compared between the two groups. At phylum level, *Firmicutes* and *Bacteroidetes* were dominant in both groups (Figure 5A). The ratio of *Firmicutes* to *Bacteroidetes* and the relative abundance of *Firmicutes* and *Tenericutes* were observably decreased by curcumin supplementation (Figure 5C–F). At the genus level, the relative abundance of *Bacteroides*, *Faecalibaculum*, *Phascolarctobacterium*, *Parasutterella* and *UBA1819* were notably enhanced (Figure 5B,G,I,J,L,M), while the abundance of *Lachnospiraceae_NK4A136_group*, *Oscillibacter* were observably reduced by curcumin treatment (Figure 5H,K).

### 3.5. Curcumin Up-Regulated the Expression of Liver CYP7A1 in HFD-Fed Hamsters

**Figure 6:**
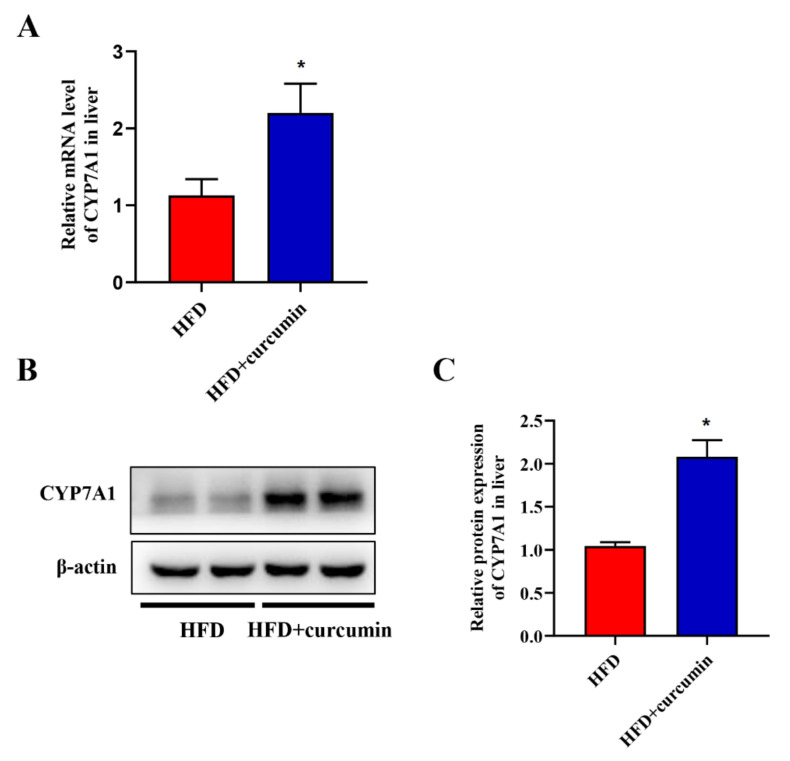
Effects of curcumin on liver CYP7A1 expression in HFD-fed hamsters. (**A**) The mRNA expression of CYP7A1 by RT-qPCR assay; (**B**) The protein expression of CYP7A1 by Western blotting assay; (**C**) Grey-scale quantitative analysis of Western blotting. Data are expressed as mean ± SEM (*n* = 8), * *p* < 0.05 compared to HFD group. CYP7A1, Cytochrome P450 family 7 subfamily A member 1.

The expression levels of CYP7A1 in the liver was evaluated through Western blotting and RT-qPCR assay. Curcumin significantly up-regulated the mRNA expression levels of CYP7A1 in hamsters fed an HFD (Figure 6A). The results of Western blotting were consistent with those of RT-qPCR (Figure 6B,C).

### 3.6. The Correlations between the Gut Microbiota and Biochemical Markers

**Figure 7:**
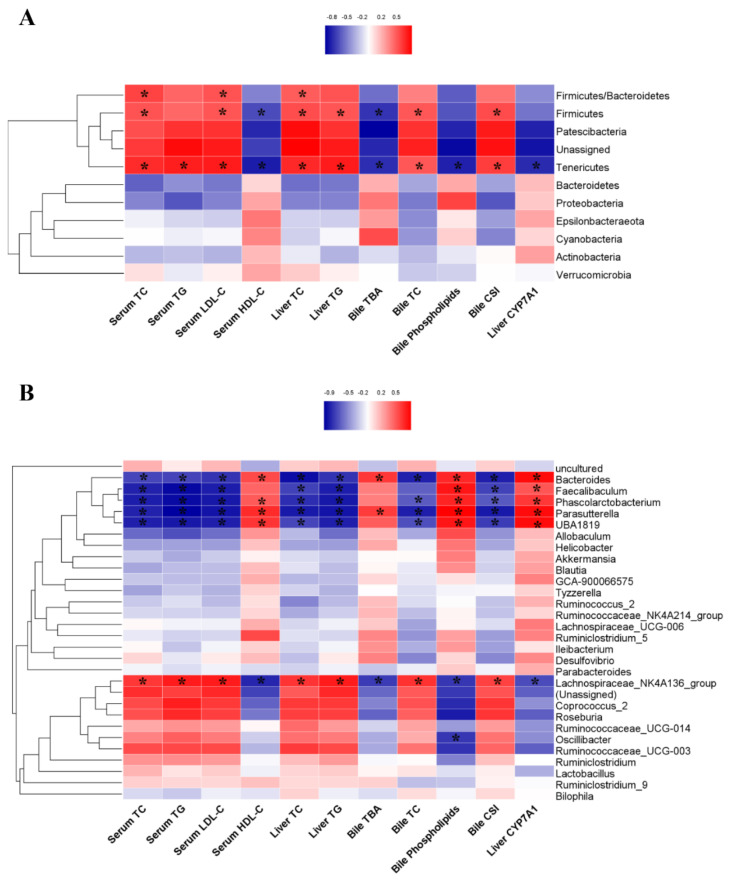
Spearman correlations between gut microbiota and biochemical markers. (**A**) Spearman correlation analysis at phylum level; (**B**) Spearman correlation analysis at genus level. Red color indicates a positive correlation between gut microbiota and biochemical parameters, while blue represents a negative correlation, and the darker the color the stronger the correlation. *n* = 6, * *p* < 0.05 significant correlations.

Spearman correlation analysis was used to explore the potential relationship between the relative abundance of gut microbiota and serum, liver, and bile biochemical markers (Figure 7A,B). Analysis at phylum level showed that *Firmicutes* was positively correlated with serum TC and LDL-C levels, liver TC and TG contents, bile TC levels and CSI, and negatively correlated with serum HDL-C and bile TBA levels. The correlation between *Tenericutes* and these indicators was opposite to that of *Firmicutes*, and *Tenericutes* presented a negative correlation with bile phospholipid concentration, liver CYP7A1 protein expression level and a positive correlation with serum TG level. The ratio of *Firmicutes* to *Bacteroidetes* had a positive correlation with serum TC and LDL-C levels and liver TC content (Figure 7A).

The heatmap at genus level indicated that *Lachnospiraceae_NK4A136_group* was positively correlated with serum TC, TG, LDL-C levels, liver TC and TG levels, and negatively correlated with serum HDL-C level, while *Bacteroides*, *Faecalibaculum*, *Phascolarctobacterium*, *Parasutterella* and *UBA1819* exhibited a completely opposite effect. Furthermore, *Lachnospiraceae_NK4A136_group* had a positive correlation with bile TC level and CSI, and a negative correlation with bile phospholipids level and liver CYP7A1 protein expression level. Conversely, *Bacteroides*, *Faecalibaculum*, *Phascolarctobacterium*, *Parasutterella* and *UBA1819* were negatively correlated with bile TC level and CSI, and positively correlated with bile phospholipid concentration and liver CYP7A1 protein expression level. Furthermore, *Lachnospiraceae_NK4A136_group* had a negative correlation, while *Bacteroides* and *Parasutterella* had a positive correlation with bile TBA concentration (Figure 7B).

### 3.7. Curcumin Reduced Intestinal Cholesterol Absorption in HFD-Fed Hamsters and Caco-2 Cells

**Figure 8:**
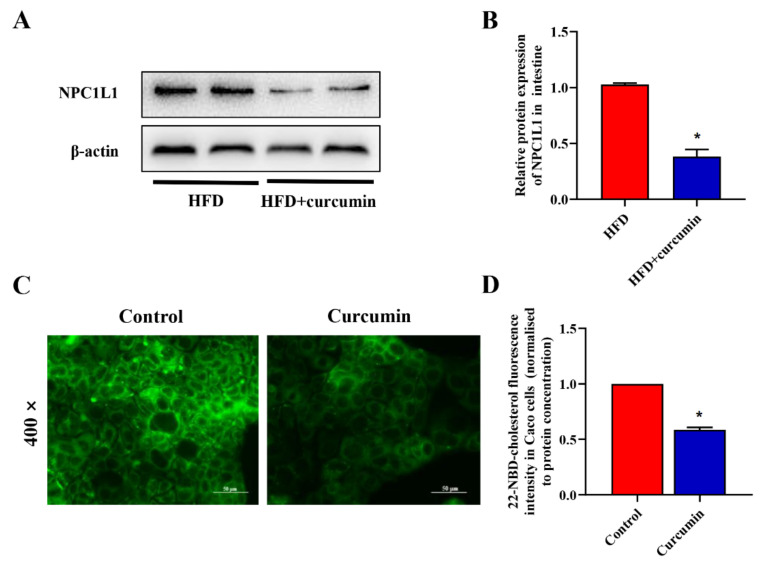
Effects of curcumin on intestinal cholesterol absorption in HFD-fed hamsters. (**A**) The protein expression of intestine NPC1L1 in hamsters by Western blotting assay; (**B**) Grey-scale quantitative analysis of Western blotting; (**C**) Representative fluorescent images of Caco-2 cells cultured with 4 μmol/L 22-NBD-cholesterol and 12 μmol/L curcumin (400× magnifications); (**D**) Quantitative analysis of 22-NBD-cholesterol fluoresce intensity. * *p* < 0.05 compared to HFD group. NPC1L1, Niemann–Pick C1-like protein 1.

To investigate the effect of curcumin on intestinal cholesterol absorption, we measured the expression of intestinal NPC1L1 protein in hamsters and cholesterol absorption in Caco-2 cells. Curcumin significantly down-regulated the protein expression level of NPC1L1 in the intestine of HFD-fed hamsters (Figure 8A,B). Furthermore, curcumin significantly reduced the cholesterol uptake in Caco-2 cells (Figure 8C,D).

## 4. Discussion

This study demonstrated that curcumin supplementation significantly improved biliary cholesterol supersaturation and decreased serum and liver cholesterol levels, thereby reversing the appropriate condition for cholesterol gallstone formation in hamsters fed an HFD. We further found that curcumin reshaped the gut microbiota composition by decreasing the richness and diversity of gut flora and altering the relative abundance of bacteria at different levels, particularly those bacterial genera associated with SCFA production and BA metabolism. There was a significant correlation between significantly different bacteria and biochemical markers, especially CSI, suggesting a potential role of gut microbiota dysbiosis in the pathogenesis of cholesterol gallstones. In addition, we found that curcumin could improve cholesterol levels through down-regulating the expression of intestinal NPC1L1 in hamsters and reduced cholesterol absorption in Caco-2 cells. Our results indicated that curcumin treatment was an effective nutraceutical for gallstone diseases and the underlying mechanism might be that curcumin modulated gut microbiota and cholesterol absorption, improved the metabolism of cholesterol and BAs, and thus reduced the formation of cholesterol gallstones.

Curcumin is the natural extract of dry rhizome of the plant turmeric. It has been considered as a golden dietary choice for the prevention of many diseases due to its anti-inflammatory, antioxidant and cholesterol-lowering effects [15,16]. In addition to the above, dietary curcumin has been reported to have a great anti-lithogenic influence. Existing studies have shown that curcumin in dietary spices noticeably reduced the incidence of cholesterol gallstones and reversed the LD-induced increase in bile CSI and TC levels and the decrease in bile TBA and phospholipids levels in mice [17,22]. Consistent with published articles, we found that curcumin treatment significantly reduced bile CSI and TC levels as well as increased bile TBA and the phospholipid contents in hamsters fed an HFD. CSI is the most reliable indicator of cholesterol crystallization in the gallbladder, and when the metabolism of BAs is disturbed or the TC content is too high, supersaturated cholesterol crystallizes into stones [23]. Our research showed that curcumin significantly decreased bile CSI, suggesting the potential of curcumin to reverse the state of cholesterol supersaturation and reduce the formation of cholesterol gallstones. The cholesterol-lowering effect of curcumin is believed to be responsible for its anti-lithogenic effect as gallstones were closely related to abnormal cholesterol metabolism [17,19,22]. Our previous studies have proved that curcumin could maintain cholesterol homeostasis by inhibiting cholesterol absorption in the gut [16,20]. Additionally, in this study, we obtained similar results that curcumin down-regulated the expression of intestinal NPC1L1 protein, which is responsible for promoting cholesterol absorption, so that serum, liver and bile TC levels in hamsters were markedly decreased by curcumin treatment in hamsters fed an HFD. We further found that curcumin significantly reduced the absorption of 22-NBD cholesterol by Caco-2 cells. Consistent with our study, Shen et al. reported that phytosterols could prevent cholesterol gallstones by inhibiting cholesterol absorption through down-regulation of intestinal NPC1L1 expression as excessive cholesterol accumulation is a prerequisite for the stone formation [24].

Researchers demonstrated that curcumin shows prebiotic potential because it is resistant to digestive juices in the gastrointestinal tract and can be selectively exploited by probiotics (health-promoting bacteria) [21], thus maintaining intestinal microflora homeostasis, promoting intestinal health and improving metabolic disorders [21,25]. In this study, curcumin supplementation reshaped the gut composition in hamsters fed an HFD. We found that curcumin treatment significantly decreased the richness and diversity of gut flora, a result consistent with published articles [26,27], suggesting that curcumin suppressed some pathogenic bacteria induced by HFD. We observed that the relative abundance of *Firmicutes* and *Tenericutes* and the ratio of *Firmicutes* to *Bacteroidetes* were remarkably decreased by curcumin treatment, and their abundance was positively correlated with serum, liver and bile lipid levels. As a major component of the gut flora, higher *Firmicutes* abundance is closely associated with obesity [28] and the value of *Firmicutes*: *Bacteroidetes* has been reported to be positively related to energy absorption and inflammation [29]. *Tenericutes* was also found to be positively correlated with obesity and could be inhibited by curcumin in previous studies [26,30]. Interestingly, we found that SCFAs producing the bacteria genera *Bacteroides*, *Faecalibaculum* and *Phascolarctobacterium* were enhanced in the curcumin-treated group, despite *Lachnospiraceae_NK4A136_group* being inhibited by curcumin. Additionally, *UBA1819*, a genus belonging to Phylum *Ruminococcaceae* was also increased by curcumin. SCFAs derived from bacterial fermentation of dietary fiber play an important role in maintaining host metabolism and intestinal homeostasis [31]. In addition to the ability to produce SCFAs, *Bacteroides* with bile salt hydrolase (BSH) activity can also alter bile components by affecting BA metabolism [32]. The genus *Parasutterella*, promoted by curcumin in present study, also has a potential role in the metabolism of BAs. The researchers found that transplantation of *Parasutterella* altered the expression of ileal BA transporters and hepatic BA synthesis genes in mice [33]. Furthermore, curcumin reduced the abundance of genus *Oscillibacter*, an opportunistic pathogen that produced lipopolysaccharide (LPS) [34]. Excessive LPS can activate the inflammatory response, leading to cholesterol accumulation and liver injury [35].

Dysbiosis of the gut microbiota is associated with multiple diseases [36]. Although the evidence is scant, existing analysis of intestinal flora composition in patients or animal models with cholesterol gallstones suggest that gut microbiota dysbiosis is associated with cholesterol gallstones [7,37]. An investigation showed that changes in the intestinal microbiome significantly altered the accumulation of mucin gel (the nucleating matrix of gallstones) and the weight of gallbladder, indicating that alterations in the gut microbiome may influence the pathogenesis even the occurrence of gallstones [8]. In line with previous expectations, our present study observed a significant correlation between gut bacteria with a significant difference and the levels of bile components. In particular, the correlation between gut bacteria and CSI indicated that genus *Bacteroides*, *Faecalibaculum*, *Phascolarctobacterium*, *Parasutterella* and *UBA1819* may have the potential to reduce cholesterol saturation levels and inhibit gallstone formation. The role of intestinal flora in the pathogenesis of gallstones can be explained by its influence on cholesterol and BA metabolism in the host, since altered BA profile or elevated cholesterol level are known to be direct causes of gallstones. The intestinal microbiota has been observed to be involved in the regulation of hepatic synthesis and intestinal absorption of cholesterol in mice, and mice receiving fecal microbiota from humans with elevated serum cholesterol levels also exhibited high serum cholesterol levels [38]. Consistently, the results of the present study showed that the genera *Bacteroides*, *Faecalibaculum*, *Phascolarctobacterium*, *Parasutterella*, and *UBA1819* were negatively correlated while *Lachnospiraceae_NK4A136_group* were positively correlated with serum, liver and bile TC levels. Interestingly, the bacterial genera mentioned above were associated with the production of SCFAs. The SCFAs acetate, propionate and butyrate, produced by bacterial fermentation of dietary fiber, play a regulatory role in gut integrity, glucose homeostasis and lipid metabolism [39]. Several studies have indicated the cholesterol-lowering potential of dietary SCFAs, and the effect may be explained by the SCFA’s influence on hepatic cholesterol synthesis and fecal BA excretion [11,12,13].

It is worth noting that the genera *Parasutterella* and *Bacteroides* were positively correlated with the concentration of bile TBA and the protein expression level of liver CYP7A1 in this study. Previous studies have shown a correlation between *Parasutterella* and alterations in BA profiles [33,40]. Ju et al. found that transplantation with *Parasutterella* significantly down-regulated gene expressions of ileal BAs transporters and the FXR signal pathway in mice, while the mRNA level of CYP7A1, which is negatively regulated by FXR, was significantly higher after *Parasutterella* colonization [33]. Therefore, we further tested the expression of hepatic CYP7A1 and found that the CYP7A1 expression level was significantly up-regulated by curcumin administration and positively correlated with the abundance of *Parasutterella*. CYP7A1 is the rate-limiting enzyme for BA synthesis and the increased expression of CYP7A1 promotes the conversion of cholesterol into BAs [10]. *Bacteroides* plays an important role in the enterohepatic recycle of BAs for it has BSH activity, which is responsible for the deconjugation of conjugated BAs [32]. After deconjugation, the free BAs are further catalyzed by bacteria with 7α-dehydroxylation activity to produce secondary BAs, which play an essential role in maintaining bile acid pool homeostasis. It is known that BAs can act as agonists or antagonists of FXR and play an important role in glucose and lipid metabolism, and activation of FXR negatively regulates the expression of CYP7A1 and Cytochrome P450 family 27 subfamily A member 1(CYP27A1), both of which are responsible for the hepatic BAs synthesis [41,42]. The increase in *Bacteroides* may enhance BSH activity, alter the BA profile, affect the FXR signaling pathway, promote the expression of CYP7A1 and thus enhance the synthesis of BAs in the liver. Our results indicate that dietary curcumin has the potential to prevent bile cholesterol supersaturation through modulating the gut microbiota and inhibiting intestinal cholesterol absorption.

However, there are still some limitations in our study. One limitation is that only male hamsters were used in present study. As sex is known to have an impact on gall-stone formation, both sexes need to be considered in further research. Another limitation is that the role of gut microbiota in the prevention of biliary cholesterol supersaturation by curcumin needs to be validated by further studies, such as antibiotic treatment or fecal microbiome transplantation, which we intend to conduct in the future.

## 5. Conclusions

Curcumin had been shown to regulate the gut microbiota, particularly the intestinal flora affecting BA metabolism and SCFA production, and to reduce the intestinal cholesterol absorption, thus reversing bile cholesterol supersaturation in HFD-fed hamsters. Our study suggests that curcumin is an effective therapeutic method to maintain cholesterol homeostasis and protect against gallstone diseases.
